# A Community-Developed, Web-Based Mobile App Intervention Addressing Social Work and Legal Needs of Black Sexual Minority Men Living With HIV: Protocol for a Randomized Comparison Trial

**DOI:** 10.2196/19770

**Published:** 2021-01-06

**Authors:** Ayako Miyashita Ochoa, Christian Corpuz Paneda, Elizabeth SC Wu, Katherine Elizabeth Maxwell, Gerald Garth, Terry Smith, Ian Walter Holloway

**Affiliations:** 1 Department of Social Welfare UCLA Luskin School of Public Affairs Los Angeles, CA United States; 2 Arming Minorities Against Addiction and Disease (AMAAD) Institute Los Angeles, CA United States; 3 APLA Health Los Angeles, CA United States

**Keywords:** HIV, AIDS, mobile apps, African Americans, men’s health, sexual minority, treatment adherence, mobile phones

## Abstract

**Background:**

Black sexual minority men (BSMM) are disproportionately affected by HIV. Los Angeles County (LAC) carries a substantial burden of the HIV epidemic in California. Negative effects of both psychosocial and structural barriers highlight the timely need to increase HIV treatment among BSMM. Successful HIV interventions based on social media and mobile phone technology have been demonstrated. This protocol describes LINX LA, a study that tests LINX, a web-based mobile app that provides tailored social services, legal resources, and peer support for BSMM living with HIV (BSMM+) in LAC using a randomized comparison trial.

**Objective:**

During phase 1, the LINX LA study aims to engage in an iterative design process to develop the LINX App using qualitative data to inform and tailor the mobile app technology and its functionality. In phase 2 of LINX LA, we will test the efficacy of the LINX App compared with the LINX App Plus to improve HIV treatment outcomes (ie, antiretroviral therapy adherence, viral suppression) among BSMM+ in LAC by addressing social work and legal needs and developing a forum for peer support.

**Methods:**

In this study funded by the California HIV/AIDS Research Program, we will recruit and enroll BSMM+ participants (aged ≥18 years) in LAC (N=400) to participate in a 12-month study that includes access to the LINX App, which provides a forum for peer support and tailored content aimed at improving the use of social and legal resources. All participants will also receive survey-based interviews at 3 time points (at baseline and 6- and 12-month intervals) and weekly text message surveys that assess medication and treatment adherence. Treatment adherence and viral suppression will be extracted from medical record data. Half of the participants will also be randomly assigned to receive 3 individualized coaching sessions (at 1-, 3-, and 6-month intervals) and the ability to directly message their coach via the LINX App. Over the course of the study, LINX App participants will receive a minimum of US $130 in cash and LINX App Plus participants will receive a minimum of US $190. We hypothesize that participants enrolled in LINX App Plus will demonstrate greater improvement in HIV outcomes compared with LINX App participants.

**Results:**

The LINX study will test the efficacy of a web-based mobile app intervention for BSMM+ in LAC (N=400). The LINX App seeks to increase participants’ knowledge of HIV; to facilitate access to necessary social and legal services, including information and referrals; and to increase social support across participants by providing a mediated forum for engagement.

**Conclusions:**

The implementation of LINX LA aims to develop and test a culturally tailored approach to improve the HIV treatment outcomes of BSMM+.

**International Registered Report Identifier (IRRID):**

PRR1-10.2196/19770

## Introduction

### Background

African American people accounted for 42% of new HIV diagnoses in the United States in 2018 [1]. Black sexual minority men (BSMM) are further disproportionately affected by HIV. BSMM accounted for 26% of new HIV diagnoses and 37% of new diagnoses solely among all sexual minority men (SMM) in the United States in 2017 [2].

In California, the annual number of HIV diagnoses increased by 0.8% from 2013 to 2017; the rate of HIV infection, however, decreased in California during the same period by 2.4% [3]. In 2017, the number of people living in California diagnosed with HIV was 135,082 individuals [3]. Within this group, 73.6% of these individuals were in HIV care and 63.3% of these individuals achieved viral suppression [3]. African American people living with HIV experienced lower rates of HIV treatment engagement in California compared with White, Latino, Asian, and Pacific Islander people [3].

Los Angeles County (LAC) carries a substantial burden on the HIV epidemic in California. In 2017, 31.6% of all newly diagnosed HIV infections in California came from LAC. Of all people living with HIV in California, 38.1% are from LAC [3]. Within LAC, African American people represented 25% of new HIV diagnoses in 2016 [4]. Between 2015 and 2016, the rate of HIV diagnoses decreased for White people in LAC; however, for African American people, the rate of HIV diagnoses increased during the same period [5]. Approximately 84% of all LAC HIV diagnoses in 2016 were among the SMM. Among those newly diagnosed SMM, linkage to and retention in HIV care was less common among African American people than among White, Latino, or Asian and Pacific Islander people [5].

Data on the continuum of care for people living with HIV in LAC in 2016 revealed that 52% of African American people were linked to care, 65% were engaged in care, 49% were retained in care, and 52% achieved viral suppression [5]. These figures, when compared with people living with HIV across California, indicate that LAC’s African American people living with HIV fare worse in terms of HIV outcomes as they have been proportionately less likely to be engaged in care and to achieve viral suppression [3].

Socioeconomic predictors, such as income status, may provide perspective on linkage to HIV care, as African American people experience poverty at a disproportionately higher rate than other racial/ethnic groups in the United States [6]. Poverty is associated with an increased risk of HIV diagnosis among some BSMM, which may also affect the health of BSMM living with HIV (BSMM+) [1] as racism continues to serve as a structural barrier to HIV services for BSMM+ [7]. Black people living with HIV who experience greater racial discrimination were less likely, as documented in prior research, to have an undetectable viral load and a high CD4 cell count [7]. African American people also expressed higher levels of medical mistrust, especially those living with HIV [8]. Higher general medical mistrust is a significant predictor of lower continuous HIV medication adherence over time [9].

Structural racism is related to the decreased likelihood of reporting antiretroviral therapy (ART) use among young BSMM+ [10]. The impact of racism on BSMM+ must be understood in the context of homophobia and HIV-related stigma. BSMM are less likely to disclose their sexual identity or sexual activity to their health care providers compared with SMM from other racial/ethnic groups [11]. These multiple layers of systemic, intersecting oppression and the resulting stigma that BSMM+ face can negatively affect health-seeking behaviors and HIV treatment engagement and highlight the need for tailored interventions for BSMM+.

### Culturally Tailored Mobile App Interventions

HIV interventions that address self-efficacy and resilience are effective in promoting health behaviors [12]. Previous studies highlight strengths-based approaches as appropriate for increasing the use of HIV services by recognizing how BSMM+ overcome challenges to care [12]. Interventions that focus exclusively on risk highlight what is lacking and reinforce harmful stereotypes about individuals facing overlapping stigma [12]. In addition, social support networks are vital to sustain healthy behaviors among BSMM+. HIV interventions that facilitate peer social support build resilience through avenues of emotional and informational support. These social exchanges are opportunities for BSMM+ to empower each other, encourage healthy behaviors, and provide information on HIV-related resources. Such interventions promote resilience processes to combat the negative effects of multiple stigmas, including those related to racial minority identity, sexual minority identity, and HIV status [12,13].

The use of social media and mobile phone technology have been proven to be effective avenues for HIV intervention [12,14,15] because they are widely available and acceptable methods for HIV intervention [16]. Mobile apps and social media platforms allow interventions and information regarding HIV to be more accessible to communities that have been deemed *difficult to reach* due to multiple stigmas [14]. Although these platforms are virtual, community partnership remains essential to maintain sustained engagement and effectiveness as collaboration ensures tailored messaging for key populations such as BSMM+ [12]. In addition, online spaces facilitate social connectivity while giving BSMM+ the ability to remain anonymous, should they choose [12].

Existing technologies, including HIV-focused mobile apps, are available to the public; however, usage remains to be low [17,18]. Technology created to address the needs of people living with HIV focuses specifically on HIV education, access to services (eg, HIV testing, linkage to care), maintaining communication between the patient and provider to support adherence and retention in care, and to support the work of providers [19]. Through its formative work, the LINX web-based mobile app (LINX LA) study identified a gap in existing mobile apps. No health mobile apps at this point focus on the complex structural and psychosocial barriers that BSMM+ face to improve HIV outcomes, including upstream factors such as housing and homelessness, lack of access to public benefits, and other social and legal needs. This study aims to test the efficacy of a web-based platform designed by and tailored for BSMM+ that provides social and legal resources as well as a forum for peer support. First, we hypothesize that participants in the LINX App Plus condition will demonstrate improvement in HIV outcomes as compared with those in the LINX App arm of the study. Second, we hypothesize that by offering social and legal services case management, the study could address 2 primary issues faced by many BSMM+—housing and financial instability.

## Methods

### Overview

The proposed study is a randomized comparison trial of a web-based mobile app titled *LINX*. Participants will be randomly assigned to either the intervention (ie, treatment group) or the comparison group. Those in the intervention group will receive the app and access to a LINX Coach (*LINX App Plus* condition) and those in the comparison group will receive the LINX Mobile app only (*LINX App* condition). Participants in both arms of the study will remain on the mobile app for 12 months. The overarching goal of the study is to evaluate the efficacy of LINX App Plus compared with LINX App to improve HIV treatment outcomes (ie, ART adherence, viral suppression). The research team did not register this study at ClinicalTrials.gov because it did not involve randomization to a true control group. Owing to ethical concerns about withholding the mHealth intervention, all participants received the app; half were randomized to receive coaching.

### Target Population

**Figure 1:**
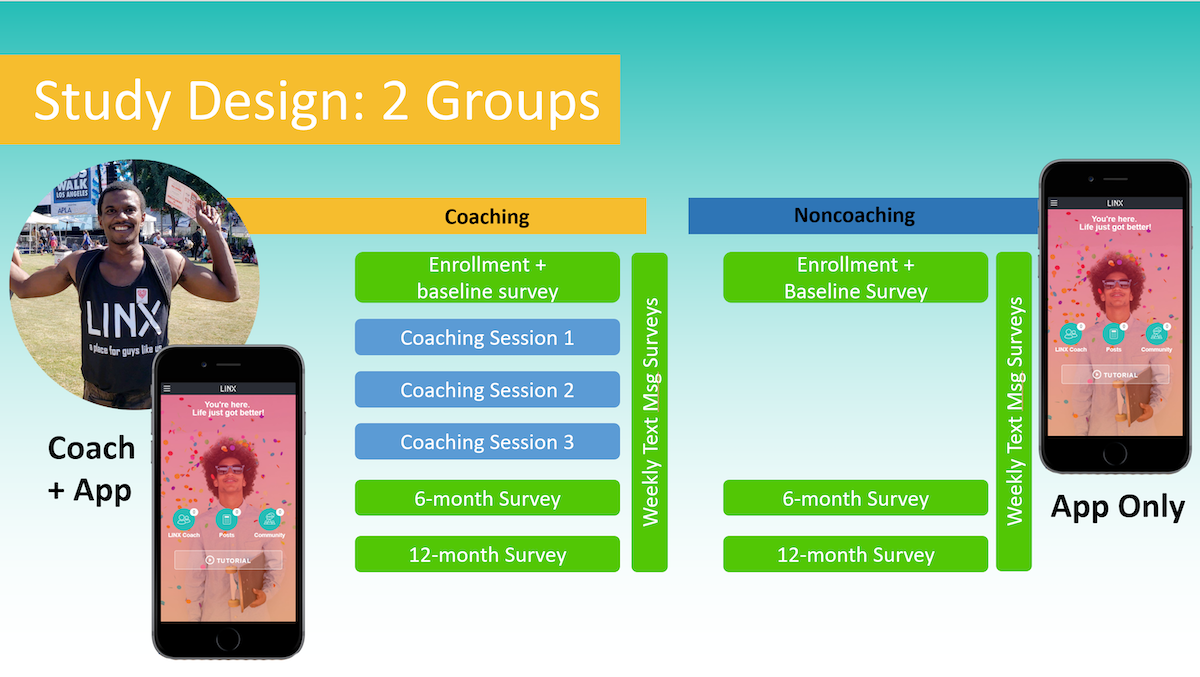
Study design of the LINX study.

The goal of the study is to recruit a minimum of 400 participants for the study. Half of the sampling group (200 individuals) will be randomly assigned to the LINX App condition and the other half will be assigned to the LINX App Plus condition (Figure 1). To be eligible for the study, participants must be aged at least 18 years old or older and must be male, Black or African American, and gay, bisexual, or other sexual minority. Participants must also own a smartphone, be living with HIV, reside in LAC, and be able to provide informed consent.

### Mobile App Development

In phase 1, we conducted qualitative interviews with BSMM+ in LAC focused on technology use patterns, HIV diagnosis and engagement in care, and social and legal service needs. Using these data, we worked with a private sector technology partner, Philosophie Group Inc [20], to engage in an iterative, user-centered design process to develop the LINX App. Throughout the intensive 6-week design process, we developed an interactive prototype and conducted moderated interviews with BSMM+ (n=20) and HIV service providers (n=11). Participants tested the functionality of a paper prototype of the LINX App through moderated interviews and to evaluate the prototype according to user experience principles (eg, content and delivery of content is useful, findable, accessible, desirable, usable, and credible). The phase 1 design process ended with a 1-week pilot test of the LINX App with BSMM (n=14), including members of the LINX LA Study Community Advisory Board (CAB).

**Figure 2:**
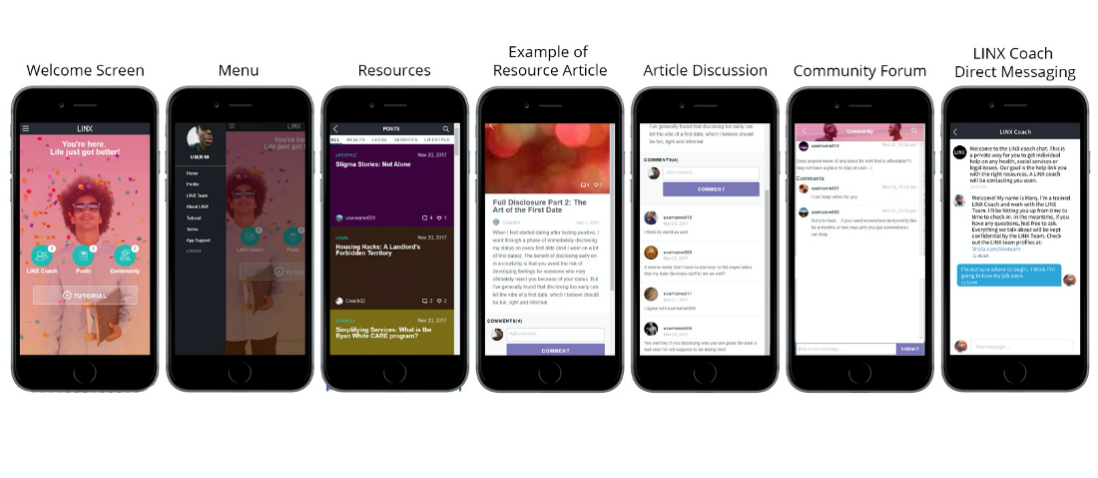
LINX App user interface.

Study staff developed a library of content including over 1000 informational posts in areas of need identified during phase 1 and issues raised during moderated interviews. The 6 main categories include health, fun, legal, relationships, services, and housing with a focus on sharing information related to HIV knowledge, HIV-related services in the community, legal rights and resources available to people living with HIV, and social services and programs. Users can also create their own content on the app, including both creative and informational posts. With the ability to post photos and videos using YouTube and Vimeo platforms, posts have the potential to be dynamic and engaging. Examples of the app interface with mockup text are shown in Figure 2.

### Recruitment

Recruitment strategies will include the following efforts (Textbox 1).

**Recruitment strategies.**

Meetings with key stakeholders

- Attend in-person meetings with key collaborators in the community including Public Health Departments, community clinics, Los Angeles County (LAC) HIV Commission, and other health care and social service providers serving Black sexual minority men (BSMM) in LAC
- Community partners, stakeholders, researchers, and staff will test and review the mobile app live and distribute study materials to their networks
- Due to the COVID-19 pandemic, our meetings will be held on the internet using the Zoom Video Communications platform

In-app referral program

- LINX LA has a built-in referral function to allow participants to refer people directly from the app
- Once a referral is made, LINX staff will be notified to follow up
- Participants will receive a US $20 Amazon e-gift card for every person successfully referred into the study

Referral program

- Noninvestigator health care, social and other service providers, and community members will be able to refer others into the study
- Referrers are sent a referral code, information about the study, a suggested pitch they can use to talk about the study, and a digital referral card to post on the internet
- Referrers will receive a US $20 Amazon e-gift card for every person successfully referred into the study

In-person outreach events

- Staff will distribute flyers and business cards for the study at locations including community events tailored to attracting BSMM, clinics and health centers, bars and clubs, retail businesses, community forums, social service organizations, and other community-based service providers
- Owing to the COVID-19 pandemic, all in-person outreach events will be put on hold; instead, all outreach will be conducted via phone, social media, and email

Web-based promotion

- Participants will be recruited through web-based methods using targeted advertising on social networking sites (eg, Facebook.com, Twitter.com, SCRUFF.com, Grindr.com, Craigslist.org, Jack’d [Jackd.com], Bareback Real Time Sex [BarebackRT.com], Black Gay Chat Live [BGCLive.com]) in addition to direct email campaigns
- Staff will engage social media influencers, people with a large number of social media followers, to help widen the reach of our web-based promotion

### Community Engagement

During phase 2 of the LINX study, researchers and study staff will engage in a range of collaborative activities to ensure that research activities are responsive to and understand community needs, views, and expectations.

#### APLA Health: Community-Based Program Implementation

The LINX study will strategically subcontract with APLA Health, a social service organization with over 35 years of work serving LA communities affected by HIV/AIDS, to implement the LINX LA study’s coaching activities. The LINX Coach will be hired from within the community and trained by and embedded within APLA Health’s well-established outreach, prevention, and social services department to ensure cross-training and resource sharing between researchers and community partners. This collaboration will enable underresourced participants to access APLA Health’s full range of in-house wraparound services—social support groups, housing navigation, mental health, benefits counseling, and health care services. APLA Health provides essential services, so their full range of services is still available (albeit some services are limited to specific locations) during the COVID-19 pandemic.

#### Arming Minorities Against Addiction and Disease Institute: Community-Driven Capacity Building and Social Events

The research team will partner with the Arming Minorities Against Addiction and Disease (AMAAD) Institute, a social services organization serving lesbian, gay, bisexual, transgender, and queer (LGBTQ) people of color in South Los Angeles. Since 2014, AMAAD developed a community-driven project to empower young LGBTQ African American people to share personal experiences and life skills through art. This partnership will accomplish several goals: (1) generate culturally appropriate content for the LINX App; (2) create and sustain social support to address isolation and disconnectedness among BSMM+; (3) identify and train BSMM+ *study ambassadors* to talk about the study and recruit peers into the study; and (4) build individuals’ capacity in editorial writing and content creation. During the ongoing COVID-19 pandemic, many AMAAD Institute programs and services, such as support groups, capacity building workshops, and training, have moved to a web-based conferencing platform (ie, Zoom Video Communications Inc).

#### CAB: Input and Feedback

The LINX CAB, made up of community members, community organization partners, and social service providers, will be established at study inception and meet on a quarterly basis to provide feedback and input on study materials, procedures, recruitment strategies, receive updates about the study, and solve implementation challenges. CAB members primarily include BSMM, including individuals living with HIV. During the COVID-19 pandemic, CAB meetings will be held on the internet via the Zoom Video Communications conferencing platform.

#### Community Outreach and Engagement: Resource Gathering and Recruitment

LINX researchers and study staff, which include community members with substantial community organizing and networking experience engaged in the LINX phase 1 formative work, will make efforts to engage with community-based organizations, clinics and health centers, HIV service providers, stakeholders, and community venues that serve or represent LGBTQ African American people across LAC. Activities will include resource sharing and cross-promoting programs and services via LINX LA–branded social media. By regularly attending resource fairs, provider network meetings, and other community meetings, LINX LA study staff will access up-to-date information on social service programs, which will facilitate effective linkages for LINX LA study participants. During the ongoing COVID-19 pandemic, in-person attendance of resource fairs and meetings have transitioned to web-based conferencing platforms.

#### Online Community Engagement: A Social Networking Extension of LINX

The LINX LA study team developed and maintained several LINX LA–branded social media accounts on platforms (eg, Twitter, Facebook, Tumblr, and Instagram) that are popular with users that match the study’s target demographic. The LINX LA study’s presence on social media serves to add to and amplify Black queer voices in the media and in the community and, in doing so, will assist the research project by providing another accessible online space for LINX App and non–LINX App users to engage followers interested in LINX LA’s curated feed who may also be interested in the LINX LA study. Growing a social media following that allows LINX LA to reach more users via followers’ networks goes beyond what is possible with traditional recruitment methods.

Words used to describe the LINX LA social media brand, developed in consultation with the LINX LA CAB, include *young, hip, Black, queer, empowering*, and *positive*, and LINX LA features original and shared content that reflects those themes and messages. Content will include posts about Black queer voices in popular culture, inspirational quotes, monthly themes, community resources, upcoming events, music playlists and videos, and articles about Black queer experiences and experiences of people living with HIV. To enhance reach and effectiveness, the LINX staff will use social media best practices, and analytics will be used to assess the effectiveness of various strategies.

### Enrollment Process

To see if an individual is eligible to enroll, potential participants must first complete a screening that can occur via email, in-person, by phone, or on the web. During the screening, LINX LA study staff will collect information regarding name or alias, age, gender identity, sexual orientation, HIV status, and race and ethnicity. The study staff then schedules in-person enrollment appointments via the participant’s preferred mode of contact. During enrollment, participants are screened for consent and asked to provide additional consent for study staff to access medical records, to provide HIV verification, and to complete a baseline assessment. During the ongoing COVID-19 pandemic, the research staff are not engaging in any in-person activities until the university and the institutional review board (IRB) that oversees the study lift restrictions related to in-person human subjects research. All enrollment activities will be conducted over Zoom Video Communications or by phone.

### Onboarding

After enrollment, participants were assisted by study staff to download the LINX App. Study staff will inform participants of the duration and compensation schedule for the study. Once the app is downloaded, the participant will be instructed how to log into the app and navigate the user interface based on the arm of the study to which the participant is assigned. Participants will be instructed on how to create a post, and the terms and conditions of use will be reviewed in detail.

### Coaching Sessions

Participants are randomly assigned to one of 2 conditions. Those assigned to LINX App Plus have access to a LINX Coach. The LINX Coach delivers 3 manualized intervention sessions via phone calls during the first 6 months of the study (ie, 1 session every other month). These sessions are adapted from a manualized intervention to promote HIV medication adherence developed by Wagner et al [21]—the adherence readiness program.

On the basis of the information, motivation, and behavioral skills model of behavior change, the 3 coaching sessions are conducted over the phone. Using additional counseling techniques based on motivational interviewing, the LINX Coach conducts 3 sessions approximately 2 months apart. Each session includes learning objectives, described in detail below. During these sessions, the LINX Coach helps to identify any social and legal needs the participant may have, provides the participant with social support and needed referrals to legal and social service providers throughout the LAC, and helps motivate participants to identify and pursue their own HIV treatment and adherence goals. The LINX Coach follows-up with participants to ensure that linkages to services have occurred. The LINX Coach also maintains regular contact with participants via 2-way messaging in the LINX App.

In session 1, learning objectives include being able to identify, with LINX Coach support, outstanding social work and legal needs and to discuss HIV treatment engagement issues that the participant may be experiencing. These objectives are achieved by discussing the intervention and expectations of the participants, the participants’ attitudes, beliefs, and goals toward treatment engagement and addressing issues discussed with the LINX Coach that are within the scope of the LINX intervention through linkage to social and legal resources.

In session 2, learning objectives include developing problem-solving skills, where the LINX Coach helps participants apply those skills to the social work and legal needs the participant may be experiencing. The LINX Coach also offers support for the participant to improve and/or maintain continued treatment engagement by addressing issues identified by the participant in session 1. This work is achieved by directly identifying barriers to engagement in care, outlining a strategy for success, and developing strategies to improve social support.

In session 3, the LINX Coach delivers the third and final coaching session. The learning objectives for this session include the participant identifying their own successful strategies for treatment engagement and troubleshooting, with help from the LINX Coach for any remaining social work and legal needs. The participant will design a plan for ongoing treatment engagement. Success in the third session is defined by the participants’ ability to both think and act independently as it relates to the identified social work, legal needs, and treatment engagement issues.

### Weekly Medication Adherence Assessments

Brief text message surveys will be distributed to all study participants to capture weekly medication adherence. The survey comprised 1 to 3 questions asking about adherence in the prior week and, if a participant reports missing their medication, a query about why they may have missed it. Participants who complete the 2-minute survey will earn 1 entry into the monthly raffle for a US $100 e-gift card. Individuals can choose not to participate or to opt out of the survey.

### Incentives

Participants in the LINX App condition will receive up to US $130 in cash, e-gift cards, or web-based cash payment (ie, PayPal Holdings Inc, Venmo LLC, and Cash App by Square Inc). Participants will receive US $30 for the baseline assessment, US $40 for the 6-month posttest interview, and US $60 for attending the 12-month follow-up interview. LINX App Plus participants will receive the same incentives as those in the LINX App condition including additional incentives for each completed coaching session. Participants in the LINX App Plus condition can receive up to US $190 in cash, e-gift cards, or web-based cash payment, as they receive a US $20 incentive per coaching session completed.

#### Discharge From the Study

Participants will be discharged from the study after completing their 12-month follow-up interview. After discharge, all individually identifiable data related to the participants will be destroyed.

### Regulations and Ethics

The research and ethics presented in this study were reviewed and approved by the North Campus IRB of the University of California, Los Angeles (UCLA; IRB#17-001615) with UCLA acting as the IRB on record for all LINX community partners. Additional terms to ensure confidentiality were provided to the participants. These terms include protection for all study-related data obtained by all LINX LA study staff, which includes employees, contractors, volunteers (paid or unpaid), and other professionals staffing the project.

Technical concerns related to data privacy were considered throughout phase I during the app development process. The mobile app is housed by a Health Insurance Portability and Accountability Act–compliant hosting platform often used by health care entities. In collaboration with the UCLA Information Technology and UCLA Compliance departments, considerations related to data storage and data in transit were considered, including the institution’s legal and ethical duties related to the handling of personally identifiable information. User terms for the mobile app were drafted to include specific guidelines on sharing data and personal information and the particular limitations to privacy for individuals who disclose information on the LINX LA app. Users are instructed to select a display name, different from their legal name, and advised not to include full facial photos, phone numbers, and email addresses to protect their identity. Procedures and protocols are established to address participants that may require intervention under the law (eg, participants expressing suicidal ideation) and participants who may be engaging inappropriately on the LINX App.

### Primary Outcomes

The primary outcomes that the study will measure include the degree to which using the LINX App may impact medication adherence and viral suppression. These primary outcomes were assessed through self-reported data verified through an independent medical chart review. The study will measure how outcomes differ between the LINX App and LINX App Plus conditions and whether the intensity of use of the platform (ie, intervention dosage) is associated with these outcomes.

### Secondary Outcomes

The overarching framework that informs the study is a social determinant of health model, where we recognize that structural factors (eg, housing instability and poverty) are upstream factors influencing individual-level health behaviors. The information, motivation, and behavioral skills model is the mechanistic model of behavior change. We posit that in the short term, the LINX App will serve to increase participants’ HIV knowledge and help create connections among the participants and connections to existing community-based resources. This may impact both treatment adherence and address issues related to social isolation and stigma. For participants in the LINX App Plus arm of the study, we hypothesize that by offering social and legal services case management, the study could address 2 primary issues facing many in the target population—housing and financial instability.

### Assessment Measures

The baseline, 6-month, and 12-month surveys comprised 12 topic areas (Table 1).

**Table 1:** Primary and secondary outcomes of the study.

Outcomes		Measures		Variables	
**Primary**					
	Technology use		Multiple items		Likert scale, range 1-5
	Medication adherence		Self-reported medication adherence; HIV medical record data		Dichotomous; Yes or No HIV medication adherence or HIV-associated medical appointment
	Viral supression		Viral load and CD4 counts		Continuous
**Secondary**					
	Unemployment		Unemployed, part-time, or full-time		Nominal
	Unstable housing		Homeless, marginally housed, or housed		Nominal
	Access to health care provider		Multiple items		Nominal and multiple dichotomous items
	Social support		Multidimensional scale of perceived social support		Continuous
	Connectedness to gay community		Items adapted from Healthy Young Men study		Ordinal
	Peer norms for HIV treatment		Perceived peer norms scale		Continuous
	Sociodemographic		Multiple items		Nominal and integer
	Victimization and discrimination		Victimization and discrimination questionnaire		Nominal
	Ethnic identity		Multigroup ethnic identity measure revised		Likert scale, range 1-5

### Measuring and Maximizing Engagement

User data will be measured using metrics such as the number of logins and the amount of time spent on the app, posts created by participants, comments posted, and the messages transmitted between the LINX Coach and participants. Change indicators will also be measured through text message surveys administered weekly, in-person assessment surveys, or via Zoom Video Communications or phone calls at 3 points over the year (baseline assessment, posttest survey at 6 months, and follow-up survey at 12 months after enrollment) and assessments of HIV-related clinical outcomes.

### Statistical Analyses

Statistical methods, models, and procedures will be selected according to the research hypotheses being tested and the types of measures involved. We will set α<.05 as the level for statistical significance but will use appropriate corrections for multiple comparisons (eg, Bonferroni and Hochberg correction) to ensure that the actual overall effective type 1 error remains at a .05 level for the tests to be conducted. The frequency and patterns of missing data were carefully evaluated. To avoid potential bias resulting from missing data, imputation (eg, hot deck or multiple imputation) will be conducted, and statistical significance tests and modeling will be conducted with and without the imputed values. The findings from these analyses will be compared, and any difference will be evaluated. Potential nonlinear relationships between continuous predictors (eg, participants’ age) and outcomes (eg, CD4 count, viral load) will be evaluated through spline functions (eg, cubic spline) [22].

### Planned Analytic Approach

Descriptive statistics of all measures involved (background characteristics, potential moderators and mediators, and outcomes) will be calculated to inform the bivariate analyses and subsequent statistical modeling. On the basis of the nature of the key outcome measures (continuous, categorical, or binary), we will use a full spectrum of analytical approaches to evaluate the data including analysis of variance, analysis of covariance, linear regression, and logistic (binary or ordinal) regression. With 3 time points, repeated-measures missed effect models will also be applied to model the global fixed effect as well as individual participants’ random variation. Our approach to modeling will be to first examine the raw associations with bivariate analyses and then the adjusted and controlled association. Confounding relationships are evaluated through changes in the regression coefficient. The final models will be selected based on goodness-of-fit measures, such as r-square, likelihood ratio, c-statistics, and analysis of covariance, a penalized likelihood. Estimates of effect will be presented with associated 95% confidence limits and associated *P* values.

Preliminary analyses will describe associations between potential predictor variables between both arms of the randomized controlled trial. The initial focus will be to understand the associations between predictors and specified outcomes and to identify confounding or co-linearity that may impact later analyses. We will also describe correlations across various domains to inform the development of our final comprehensive model. We will develop base models predicting engagement in HIV care from demographic predictors (ie, age, education, income, housing status) to identify demographic variables that will be controlled for (as confounding variables or effect modifiers) when examining associations between intervention effects and outcomes. Analyses will be performed to determine robustness and to understand potential confounding or collinearity between predictors. Testing of significance for independent variables will include Wald chi-square tests for continuous or dichotomous variables and/or global goodness-of-fit tests if polytomous categorical variables are regressed. Estimates of effect will be presented with associated 95% confidence limits and associated *P* values.

### Sample Size Calculation and Power Analysis

Our proposed sample size is based on a comparison of the main outcome of linkage to HIV care between the study arms of the LINX App and LINX App Plus conditions based on a priori logistic regression sample size calculations in PASS 2008 software. Calculations assume linkage-to-care base rates across conditions of 70% based on 2013 estimates of linkage to care within 3 months of an HIV diagnosis for African American people. We also assume a type 1 error of .05 and type 2 error of .2 (or power 80%). Our targeted enrollment of 200 participants in each condition will give us enough power to detect a difference in HIV linkage between LINX App and LINX App Plus arms as low as 12.5%. In addition to comparisons between the 2 study arms as a dichotomous measure, we will also compare linkage to care and other outcomes by intervention dosage as a continuous measure. Comparisons of continuous measures often increase power analyses relative to comparison of dichotomous measures; this comparison is similarly anticipated to do so in our study.

## Results

### Expected Outcomes

The strengths of this trial are rooted in its work to address the effect of HIV among a group of individuals hit hard by the epidemic. In LAC, BSMM are facing an increase in the numbers of new HIV diagnoses, and linkage to care has historically been a specific challenge for these men. The high level of need provides an opportunity to make a measurable impact through this trial. The intervention’s focus on social services and legal needs is distinct. Although structural barriers are resistant to change and require sustained efforts, including advocacy at a policy level, specific assistance provided to ameliorate the effects of these barriers is achievable. This work will be a defining hallmark of the intervention. Finally, the use of mobile technology to foster a voice and a collective identity across BSMM+ and as an avenue for accessing resources and promoting social support, although not new, is still a strategy worth exploring.

The LINX LA study’s contribution will be to increase the understanding of how to develop technology for and by the communities targeted by an intervention and how to establish programmatic structures to ensure that the platform maintains a strengths-based lens in lifting voices from the same community. Investment in such strategies has the potential to improve ART adherence and increase viral suppression among BSMM+.

### Expected Timeline

The LINX App was completed and recruitment and enrollment for the trial started in May 2018 on a rolling basis. Owing to the outbreak of COVID-19, the collection of survey data at 3 time points over 12 months will pose a challenge. Nonetheless, the length of the study per patient will be 12 months. We expect to complete data collection 12 months after the last participant has been enrolled in the study, that is, by January 2021, and plan the dissemination of results subsequently.

## Discussion

### Limitations: Recruitment Challenges

There are potential challenges to this trial. It employs convenience sampling techniques to recruit the target population, which limits generalizability. In addition, if recruitment fails to yield the required number of participants, we may not have adequate statistical power to detect effects of intervention conditions on outcomes. To address these uncertainties, we plan to compare our sample to data from other projects focused on BSMM+ in LAC. The LAC Division of HIV and STD Programs has implemented a clinic-based medical care coordination (MCC) program to increase viral suppression (VS; <200 c/mL) among people living with HIV at high risk for poor health outcomes (eg, diagnosed with HIV in the past 6 months, on ART without VS, out of care for more than 6 months). The MCC program collects clinical data across 35 Ryan White Program–funded clinics across the county serving these populations, including those that identify with our target population. Comparing results from the study with data from participants in the county’s MCC program will help to address this gap.

Recruitment of BSMM+ may be challenging in LAC due to the sheer size of the county and the study’s eligibility criteria, requiring participants to be both living with HIV and identifying as a sexual minority. For this reason, study staff will implement a diverse set of strategies to engage varied audiences during the recruitment period, including web-based efforts and in-person efforts detailed in Textbox 1. The study will also offer participants with transportation assistance. By providing access to ride-sharing resources through Lyft Concierge services, participants will be able to meet study staff at the location of their choice. This additional support has proven effective in reducing the number of cancelled meetings between study staff and participants [18].

### Limitations: Engagement and Retention Challenges

This service should also help with a potential foreseeable challenge—the retention of participants. Study staff will develop a targeted retention plan that looks at maximizing every opportunity that the study staff may have when meeting participants. This includes walking participants through a process of updating multiple points of contact information and ensuring that participants have access to LINX LA so that efforts to promote engagement continue to reach participants. Another planned strategy includes tracking all contact with participants and identifying problems with communication at the earliest possible time point. For example, by reviewing weekly text message data, the staff can identify which participants have mobile phone numbers that may be out of service or otherwise may not be receiving study text messages.

Finally, although creative content developed by members of the community is a key strategy to driving traffic to LINX LA, the study will be challenged to command the attention of participants who currently spend time on other social media and web-based platforms. Given the significant investment in developing a mobile app for and by BSMM+, the study will need to remain nimble in its efforts to engage participants on the platform. Thus, an ongoing review of back-end user data, including analytics on usage patterns, combined with feedback provided by participants during assessments and coaching sessions must drive future strategies to retain an engaged pool of participants.

## Abbreviations

AMAAD: Arming Minorities Against Addition and Disease Institute
ART: antiretroviral therapy
BSMM: black sexual minority men
CAB: Community Advisory Board
CD4: cluster of differentiation 4
IRB: institutional review board
LAC: Los Angeles County
LGBTQ: lesbian, gay, bisexual, transgender, and queer
LINX App: LINX web-based mobile app arm
LINX App Plus: LINX web-based mobile app with LINX Coach arm
LINX LA: LINX web-based mobile app
MCC: medical care coordination
UCLA: University of California, Los Angeles
VS: viral suppression
